# Undiagnosed Accidental Blister Pack Pill Ingestion in Elderly

**DOI:** 10.7759/cureus.17167

**Published:** 2021-08-13

**Authors:** Priyanka Dwivedi, Gaurav Singh, Shahbaz Ahmad

**Affiliations:** 1 Anaesthesiology, All India Institute of Medical Sciences, Gorakhpur, IND; 2 Anaesthesiology, Baba Raghav Das (BRD) Medical College, Gorakhpur, IND

**Keywords:** blister pack pill, intestinal perforation, elderly, foreign body, accidental ingestion

## Abstract

Ingestion of foreign body is a common occurrence in children. Most of these foreign bodies pass through gastrointestinal tract without causing any symptom or complication. Sharp edgy objects have propensity to cause tear or damage to the mucosal linings of gastrointestinal tract. Here is an interesting case of unintentional ingestion of blister pack pill in an elderly, whose initial presentation was intestinal obstruction and later on developed intestinal perforation.

## Introduction

Ingestion of foreign body is a common clinical entity encountered in clinical practice. It can be accidental as in children or intentional in the mentally impaired, alcoholics, prisoners, and psychiatric patients [1,2]. Elderly population is also tended to ingest dietary foreign bodies like fish and meat bones due to poor intellectual abilities, cognition, and diminished vision. Artificial dentures and other orthodontic pieces account for most of the foreign body ingestion in elderly, whereas toys, jewelries, coins, nails, needles, pins, and small batteries are more common in children. However, most of the foreign bodies pass without any complications, and only about 1% is known to cause complications, particularly gastrointestinal perforations [2,3]. Blister pack pills (BPPs), also known as push-through pills, are very unusual to ingest as a foreign body but when ingested their sharp edges and pointed corners may cause perforation. Here is an interesting case illustrating the same, with initial diagnosis of intestinal obstruction, which later developed into perforation.

## Case presentation

A 75-year-old male presented to emergency room with dyspnea, generalized pain, and distension in abdomen for three days and inability to pass feces or flatus for two days. The patient and attendants were asked for any history suggestive of chronic abdominal symptoms like tuberculosis, loss of apatite, chronic alcoholism, previous surgery, or any history of foreign body ingestion but they denied of all. There was no history of any other chronic illness or comorbidity. On examination patient had a pulse rate 118 per minute, blood pressure 160/90 mm Hg, and respiratory rate 28 per minute with 94% saturation on room air. Abdomen was distended and generalized tenderness present in all quadrants. After initial resuscitation, radiological investigations were done, which revealed multiple air fluid levels suggestive of intestinal obstruction. Lab investigations were within normal limit except raised total leucocyte count (TLC) count of 13,900/mm^3^ and hemoglobin 9.2gm%. Since the patient was hemodynamically stable, a conservative management was decided, but after 4-6 hours, the general condition of the patient started deteriorating and decision for emergency laparotomy was made. The patient was informed and consent was taken. On exploration under general anesthesia, stomach and part of transverse colon were found densely adhered to omentum. Adhesiolysis was done along with suction of 2.5 liters of purulent fluid. Bowel was inspected but no obvious perforation was detected and surgery was completed with diversion loop ileostomy 1.5 feet proximal to Ileo-colic junction along with left flank drain. The patient was shifted to recovery intensive care unit (ICU) in intubated state.

**Figure 1:**
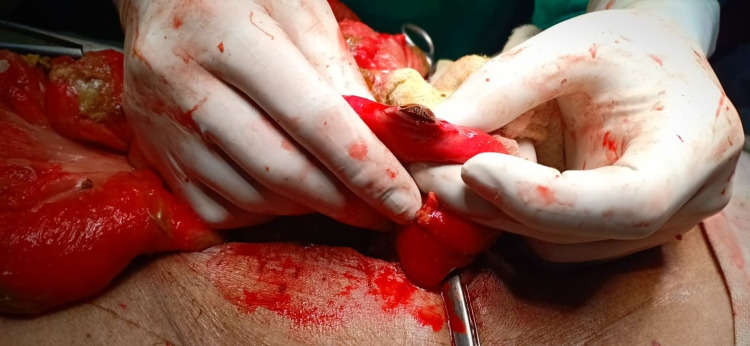
Blister pack pill peeping through the perforated bowel

On the third postoperative day bile mixed fluid was seen in the drain along with increasing TLC and abdominal distension, raising the possibility of small bowel perforation. Considering these findings, decision was taken for re-exploration under general anesthesia. On the first go, bowel examination revealed no perforation but because of the presence of bile in the drain surgeons were not convinced and palpated small bowel several times and finally traced a small, hard, and sharp object peeping through the bowel approximately 0.5 foot distal to the duodenojejunal flexure (Figure 1). The foreign body (BPP) was removed through the perforation site and resection of half foot bowel at the perforation site with end-to-side anastomosis half foot distal to duodenojejunal flexure was done. Two drains were placed on suction for 24 hours and abdominal closure was done. The patient was shifted intubated in ICU. The postoperative course was uneventful.

## Discussion

Foreign body ingestion is mostly seen in children, elderly, psychiatric patients, persons with substance abuse, professionally exposed persons like carpenters, dressmakers, and upholsterers, and sometimes as suicidal attempts. Elderly persons are at increased risk due to a number of confounding factors like poor vision, poor cognitive function leading to dependency on others, intake of higher number of drugs due to advancing age, and associated co-morbidities (polypharmacy) [3,4]. Use of artificial dentures and lack of normal palatal and gingival sensation play an important role in accidental ingestion of BPP in this population group [5]. Medicines manufactured by pharmaceutical companies are usually packaged in sets of 10 pills together in blister pack strips made up of aluminum sheets, which do not have sharp edges and corners, but when pharmacists cut these strips to give medicines according to patients' need, the cut sides of blister packs become sharp, pointed, thin, and edgy, and become a potential risk object if swallowed. Ingestion of a BPP is unusual, and its sharp and pointed edges make perforation a possible complication as they pass through the gastrointestinal tract [2,6], although few cases of obstruction have also been reported in the literature [4,7].

In this case, patient was unaware of the ingestion of BPP and so this information could not be extracted during the initial assessment. Moreover, BPP remained undetected in radiological examination and operating surgeon also missed the palpation of this sharp, edgy, and tiny foreign body during initial surgery, which rendered the initial surgery unsuccessful and finally re-explorations identified the culprit. BPPs are usually radiopaque and typically identifiable by their aluminum foil backing and plastic blister surrounding the pill along with a thin rim of air. Characteristically their lateral appearance has been compared to that of a “unidentified flying object (UFO)” [7]. Density of the pill could be extremely variable, as some pills being completely radiolucent and can potentially be seen on CT scan [8]. But many previous reports show that even CT scans (with or without contrast) also often missed such foreign bodies and re-evaluation of CT after surgical exploration correlates with the surgical finding [4].

## Conclusions

Ingestion of BPP as a foreign body is a rare occurrence and it usually leads to intestinal perforation but may present as obstruction. History taking is an important tool in making diagnosis, but it remains a challenge in elderly patients due to their poor communication, cognition, and memory. Patient's denial or ignorance about any possible ingestion of foreign body makes the diagnosis difficult; therefore, a detailed history from the relatives or primary care givers should also be always asked. Being radiopaque BPP should be identifiable in radiological examination but due to their orientation in the bowel, their presentation can be variable and often missed. Therefore, surgical exploration should be the confirmatory step in making diagnosis but it can also be missed if not done carefully. 

Due to constant aging and cumulative risk factors, elderly patients are at increased risk of accidental ingestion of foreign body; therefore, patients with such presentation need high index of suspicion and a thorough history taking along with intraoperative exploration of bowel remain a critical step in definitive diagnosis and management.
